# The New Sydenham Society

**Published:** 1859-11

**Authors:** 


THE NEW SYDENHAM SOCIETY.—This Society, under the presidency of Dr. C. J. B. Williams, is now actively engaged in the exercise of its self-imposed labors of furnishing their professional brethren, everywhere, with the works of distinguished foreign authors, and also some of the older works of their own countrymen. We wish the Society all possible success in their laudable undertaking. The annual subscription is one guinea, for which members for the present year obtain the following five octavo volumes:—

Vol. I.—*Diday* "On Syphilis in Infants and Children at the Breast." Translated by Dr. Whitley.

Vol. II.—*Gooch* "On the most important Diseases of Women and Children," with other Papers. With Wood-cuts. Prefatory Essay by Dr. Ferguson.

Vol. III.—"*Memoirs on Diphtheria.*" From various French sources. Selected and Translated by Dr. Semple. With a Bibliographical Appendix by Mr. Chatto.

Vol. IV. comprises the two Works of *Professor Schrœder Van Der Kolk*, (1st) "On the Spinal Cord," and (2d) "On the Medulla Oblongata," and "On the Proximate Cause and Rational Treatment of Epilepsy." Translated by Dr. W. D. Moore, of Dublin. With numerous Lithographs.

Vol. V. will contain Translations of the following monographs:—

1st. *Kussmaul* and *Tenner's* "Experimental Researches on the Effects of Loss of Blood in inducing Convulsions." Translated by Dr. Bronner, of Bradford.

2d. *Wagner* "On the Resection of Bones and Joints." Translated by Mr. T. Holmes. Numerous Wood-cuts.

3d. *Professor Gräfe's* Three Papers on Glaucoma, Iridectomy, etc., etc. Translated by Mr. Windsor, of Manchester.

Works in preparation for 1860:—

Vol. VI.—*Clinical Memoirs on Abdominal Tumors and Intumescence.* By Dr. Bright. Collected and Reprinted from the Guy's Hospital Reports. Edited by Dr. Barlow. With numerous Wood-cuts.

Vol. VII.—*A Year-Book for* 1859. The Year-Book is designed to form a Register in condensed abstract of all important Contributions, whether British or Foreign, in Medicine, Surgery, and their allied Sciences during the year. It will consist of five sections:—

1st. Anatomy and Physiology. Edited by Dr. Harley.

2d. Medicine. Edited by Dr. Handfield Jones.

3d. Surgery. Edited by Mr. Hulke.

4th. Diseases of Women and Children. Edited by Dr. Graily Hewitt.

5th. Forensic Medicine and Toxicology. Edited by Dr. Odling.

Vol. VIII.—*Frerich's Clinical Account of Diseases of the Liver.* Vol. I. With Wood-cuts. Translated by Dr. Murchison, Assistant Physician to King's College Hospital.

An offer on the part of Professor Simpson, of Edinburgh, to edit, for the Society, a Reprint of *Smellie's Midwifery*, with Notes, bringing the work up to the state of present knowledge, has been gladly accepted by the Council, but it is uncertain whether this book will form part of next year's issue.

*Hebra's Atlas of Illustrations of Skin Diseases.*—Conditional arrangements have been made to commence the issue of selected Illustrations from Professor Hebra's magnificent and costly Atlas; but the expense will be such that the Council cannot undertake this work unless the Society's members for 1860 shall exceed 3000.

